# Taking presentations seriously: Invoking narrative craft in academic talks

**DOI:** 10.1007/s40037-017-0366-9

**Published:** 2017-07-13

**Authors:** Kulamakan Kulasegaram, Douglas Buller, Cynthia Whitehead

**Affiliations:** 1 The Wilson Centre, University Health Network, Toronto, Ontario Canada; 2 Department of Family & Community Medicine, Faculty of Medicine, University of Toronto, Toronto, Ontario Canada

As academics, we attend a lot conferences where the majority of talks we sit through are just plain bad. Presenters ascend to the podium to read from a set of unfinished jottings, dots and half-statements scattered across randomly selected backgrounds. This is ironic because as health professions educators, we care about good communication and teaching. Many of us study how to share information and knowledge in ways that have meaning for learners. The premise of this article is that we have an imperative to become better communicators and presenters of our science. To do so, we suggest looking to the art and craft of narrative.

We can identify various reasons why academic presentations are so often poorly done. Many faculty members only receive institutional support to attend a conference if they give a presentation. Academic credit, moreover, comes from the list of presentations on one’s CV. Writing good abstracts is better value than crafting good presentations. Add the lure of a pleasing venue (Vancouver, Barcelona etc.) and our conference culture makes sense. Presentations are not serious business because it is their acceptance, not their quality, that contribute to academic advancement. Even if we have an explanation, it is still an undesirable state of affairs. We do a disservice to the people who sit, bored and distracted, through our presentations. We do a disservice to our own academic work by not taking the time to write a presentation that communicates the new knowledge we are trying to share. And for those of us who teach or work at publicly funded universities and hospitals, it is a disservice to the patients and students that we take time to travel and give dull talks or attend unmemorable ones. Too often, researchers suggest that their ideas are so complex that the audience is unlikely to grasp them. These presentations are frequently filled with overly busy charts and illegible graphs. We contend that this approach demeans the audience and does a disservice to the research. A researcher who cannot share his or her work fails.

Contrast this with the rigour and diligence with which we approach our other major communication medium: writing [1, 2]. Narrative disciplines and writers’ craft provide literature and resources to support effective academic writing [3]. Successful academic writers know that writing is an iterative and dynamic process that yields new understandings or clarifications. Lingard and others in this journal have provided extensive advice and strategies for improving writing along those lines. This lived experience is consistent with emerging evidence on the analytic dimension of writing [4, 5] and how to optimize it. It is when we respect this, the generative value of writing, that it becomes reasonable to spend as much time writing and editing a script as is possible. The all-too-common current approach of working from concepts, attempting to improvise the full text from roughly hewn notes, causes no end of challenge. It would seem absurd to submit the first draft of a piece of writing for publication, yet our audiences are expected to engage with content that could barely be considered that. In even the most visual of presentations a substantial volume of content will be delivered as text and narration. Ensuring that this text and narration are well composed will improve an audience’s potential for understanding it.

When writing our presentation it is essential to make it about more than image management, overcoming public-speaking anxieties, or the next conference in an exotic location. Crafting a good presentation is an exercise in creating meaningful connections between each of our concepts (data) [6] as well as between the audience and the content. Presentations are dependent on moment to moment interactions, so each element of the presentation must serve the dual purpose of conveying information and facilitating interaction [7, 8]. In other words, a presentation must be informative and engaging in order to be successful.

There are several approaches that can help achieve these goals. We have already mentioned the benefits of writing talks prior to presentation. Moreover, a notable amount of work has been done in the field of multimedia that takes seriously the proposition that presentations are intended to facilitate learning [8]. Models of learning such as cognitive load theory [9]offer an empirical basis for the recommendations to improve presenting. These theories are consistent with a much older body of knowledge in the narrative arts and crafts such as cinema, theatre, and storytelling.

The advice these fields offer may seem intuitive or ‘common sense’. However, as Voltaire noted, ‘common sense is not so common’ [10]. One key advice from these fields is that effective interaction and information are mutually dependent on one another. An incoherent chart or a series of bullet lists may be *informationally* rich but limit interaction. Amusing comics or photos may facilitate *interaction* but can be tangential to content. Still, these are both regular features of academic talks. One simple step to improve is to remove all of the extraneous information (and thus the extraneous load) from talks. Too often the challenge of academic talks is too much information rather than too little. If we can accept that ‘omit needless words’ is a necessary edict for good writing the presentation version would be ‘omit needless everything:’ needless bullets, needless graphs, needless pictures and needless content [11].

In place of the needless content, focus on making the talk about what is salient in the work being presented. Consider presenting one major finding and leaving minor findings for another day. Consider focusing on a novel method or unexpected insight. Then plot the most efficient route to that purpose. In other words, start at the end and work back to the beginning. A good conference presentation is one with a clear purpose and a well-marked path to that purpose. Audiences will ask for more information if they want it. They can’t, however, ask you to stop when you offer too much.

Using the lens of narrative also allows us to recast the role of the audience. Presenters often worry about the audience – in fact they worry most before they have even met anyone in the actual audience. ‘Know your audience’ is a common piece of advice but often conjures a vision of our worst critics. Not only does this increase the stress of presenting, it also encourages a repetitive and conservative approach. A different interpretation of knowing the audience is to attend to the realities of how human beings engage with information and interact with narratives. Knowing the audience means knowing the limitations of memory, attention, and perception. It is incumbent on the presenter to recognize this and present information in way that conveys the most important point of the work through making it the most engaging focus. It is not their fault if we lose them. A distracted audience is not distracted because of their shortcomings or malice. They are distracted because our presentation has fundamentally failed to provide them with what they require to engage with our content. Understanding and applying what influences attention and retention will only improve our ability to make content engaging.

We recognize the academic talk is a genre with expectations and traditions. We also strongly believe that presentation is an opportunity to use all the tools at our disposal to *teach and learn *from one another. Our commentary is offered in this spirit of teaching and learning. We too are equally guilty of some of the problematic practices we have drawn attention to. We offer these reflections as a call to improve our practice by drawing on other traditions that are readily available to us.

## Practice points

1. The act of writing a presentation can yield a clear academic presentation and provide clarity on the topic of presentation.
2. Work backwards from key message or conclusion you want the audience to understand at the talk.
3. Plot the most efficient and engaging route to this conclusion when writing your presentation. Remove extraneous information that detracts from this conclusion; focus the presentation on the salient points that lead up to your conclusion.
4. Each element of the presentation must serve the dual purpose of conveying information and facilitating engagement with the presentation. The effectiveness of conveying information depends on the level of engagement or interaction with the audience.
5. Interaction with the audience in a talk means engaging their attention and memory on the concept(s) you wish to convey.
6. You can more effectively engage with the audience by designing your talk around instructional design and information processing principles that address the audience members’ capacities for attention and memory.
7. Creating presentations is an exercise in creating meaning out of slides, words, and concepts. Revisit your talk once you have completed it and evaluate whether the meaning you want to convey is delivered effectively through the elements of your presentation.
